# Contrast-Enhanced Ultrasonography in Differential Diagnosis of Benign and Malignant Ovarian Tumors

**DOI:** 10.1371/journal.pone.0118872

**Published:** 2015-03-12

**Authors:** Jing-Jing Qiao, Jing Yu, Zhe Yu, Na Li, Chen Song, Man Li

**Affiliations:** The Second Department of Oncology, the Second Affiliated Hospital of Dalian Medical University, Dalian 116027, P.R. China; Kaohsiung Chang Gung Memorial Hospital, TAIWAN

## Abstract

**Objective:**

To evaluate the accuracy of contrast-enhanced ultrasonography (CEUS) in differential diagnosis of benign and malignant ovarian tumors.

**Methods:**

The scientific literature databases PubMed, Cochrane Library and CNKI were comprehensively searched for studies relevant to the use of CEUS technique for differential diagnosis of benign and malignant ovarian cancer. Pooled summary statistics for specificity (Spe), sensitivity (Sen), positive and negative likelihood ratios (LR^+^/LR^−^), and diagnostic odds ratio (DOR) and their 95%CIs were calculated. Software for statistical analysis included STATA version 12.0 (Stata Corp, College Station, TX, USA) and Meta-Disc version 1.4 (Universidad Complutense, Madrid, Spain).

**Results:**

Following a stringent selection process, seven high quality clinical trials were found suitable for inclusion in the present meta-analysis. The 7 studies contained a combined total of 375 ovarian cancer patients (198 malignant and 177 benign). Statistical analysis revealed that CEUS was associated with the following performance measures in differential diagnosis of ovarian tumors: pooled Sen was 0.96 (95%CI = 0.92∼0.98); the summary Spe was 0.91 (95%CI = 0.86∼0.94); the pooled LR^+^ was 10.63 (95%CI = 6.59∼17.17); the pooled LR− was 0.04 (95%CI = 0.02∼0.09); and the pooled DOR was 241.04 (95% CI = 92.61∼627.37). The area under the SROC curve was 0.98 (95% CI = 0.20∼1.00). Lastly, publication bias was not detected (*t* = −0.52, P = 0.626) in the meta-analysis.

**Conclusions:**

Our results revealed the high clinical value of CEUS in differential diagnosis of benign and malignant ovarian tumors. Further, CEUS may also prove to be useful in differential diagnosis at early stages of this disease.

## Introduction

Ovarian cancer is the fifth leading cause of cancer-related deaths among women in both developing and developed countries, and a substantial burden to global public health [1]. It is estimated that 50–60% of the deaths in ovarian cancer patients is linked to locally advanced disease [2]. Up to 10% of ovarian cancer patients are diagnosed with distant metastases, and the most common sites of organ metastases include breast, gastrointestinal tract and reproductive tract [2]. Despite the significant reduction in mortality rates of ovarian cancer patients over the past decades, the overall incidence rates continue to increase rapidly, even in countries that historically had low rates [3,4]. Large population-based studies clearly indicated that current cytotoxic chemotherapy drugs are not effective in eliminating ovarian cancers, and combination therapy with itraconazole only marginally improves patient survival [5]. Therefore, it is extremely important to find effective ways to diagnose ovarian cancers at early stages, before the disease spreads locally or metastasizes to distant organs [6]. Angiogenesis is a crucial factor in tumor cell proliferation and metastatic dissemination in various human cancers, including ovarian cancers, therefore, a reliable assessment of tumor angiogenesis and micro-vessel density (MVD) in ovarian cancers is critical for correct prognosis of patients [7,8].

Contrast-enhanced ultrasound (CEUS) is widely regarded as a reliable and convenient diagnostic imaging technique in ovarian cancer patients. CEUS, involves administration of micro-bubble contrast agents to reveal critical and sensitive information on tissue perfusion and blood flow, and is also effective in visualizing small arterial vessels less than 100 m in diameter. CEUS is considered as a relatively safe technique, and does not involve ionizing radiation or the risk of nephrotoxicity [9–12]. Unlike the color Doppler ultrasound, which is good at assessing large vascular networks in tumors, CEUS is better at interrogating tumor microvascularity, which is associated with slow blood flows and similar acoustic properties and thus, CEUS is a big advantage in diagnostics in ovarian cancers [13,14]. CEUS also significantly improves the detection of microcirculation perfusion and previous studies showed that MVD difference is invaluable in evaluating benign and malignant ovarian cancers [15]. Further, several studies observed specific CEUS enhancements in different types and stages of ovarian cancer: ring-like enhancement seen in the ovarian cystic wall and ovarian cyst papillae in benign tumors; scattered mild enhancement and branching moderate enhancement observed in solid tumors [16,17]; overall heterogeneous enhancement and rapidly increased enhancement in malignant ovarian cancer [18]. Consistent with this, several previous studies have found that CEUS can accurately distinguish benign ovarian tumor from malignant ovarian tumor [19–21], but other studies were unable to confirm the results. [22]. In order to comprehensively address this issue, we performed a systematical meta-analysis to assess the performance of CEUS in differential diagnosis of ovarian tumor mass.

## Methods

### Data sources and keywords

To identify relevant published studies that evaluated the diagnostic value of CEUS in ovarian cancer, PubMed, Cochrane Library, CNKI databases (last updated search in November 1^st^, 2013) were comprehensively searched. Keywords used included “ovarian neoplasms” or “ovarian cancer” or “ovarian carcinoma” or “ovarian tumor” or “OC” for the diagnostic factors, and “contrast-enhanced ultrasound” or “contrast-enhanced ultrasonography” or “contrast ultrasonography” or “ultrasound contrast imaging” or “CEUS” for interventions. We also manually searched the bibliographies of prominent studies to identify additional relevant studies.

### Inclusion and exclusion criteria

The studies selected for the present meta-analysis fulfilled the following inclusion criteria: (1) the studies were clinical diagnostic test; (2) the studies addressed the accuracy of CEUS in differential diagnosis of malignant ovarian cancer and benign ovarian tumors; (3) all patients diagnosed with ovarian cancer were confirmed by histopathological examinations; (4) all lesion diagnosed were histologically confirmed after CEUS; (5) published data in the four-fold (2 × 2) tables must be sufficient. Studies that did not conform to the inclusion criteria were excluded from our meta-analysis. For duplicate studies, we only choose the latest or the study with largest sample size. The PRISMA Checklist is provided as supplementary information, see S1 PRISMA Checklist.

### Data extraction

Two investigators separately collected information from eligible studies. The following data was collected: first author, publication year, study design, ethnicity, number of participant, age, gender and country, number of lesions, contrast agent and diagnostic accuracy.

### Quality evaluation

Two or more investigators assessed the methodological quality in accordance with QUADAS (quality assessments of diagnostic accuracy studies) [23]. QUADAS covers 11 domains: whether representative spectrum is included (QUADAS01); whether reference standard is acceptable (QUADAS02); whether delay between tests is acceptable (QUADAS03); whether partial verification avoided (QUADAS04); whether differential verification avoided (QUADAS05); whether incorporation avoided (QUADAS06); whether reference standard results blinded (QUADAS07); whether index test results blinded? (QUADAS08); whether relevant clinical information is included (QUADAS09); whether uninterpretable results reported (QUADAS10); whether withdrawals were explained (QUADAS11).

### Statistical analysis

To calculate the effect size for each study, the pooled statistics for specificity (Spe), sensitivity (Sen), positive/negative likelihood ratios (LR^+^/LR^−^) as well as diagnostic odds ratio (DOR), and their 95% CIs, were utilized. In order to provide adequate evidence of all enrolled studies, as well as minimize the variance of the summary, Sen, Spe, LR^+^/LR^−^, and DOR with 95%CI were calculated. Random-effect model was employed if heterogeneity existed among the studies, otherwise fixed-effects model was utilized. The summary receiver operating characteristic (SROC) curve and the corresponding area under the curve (AUC) were obtained [24]. Threshold effect was assessed using Spearman correlation coefficients. The subgroup and meta-regression analyses were also carried out to explore potential sources of heterogeneity. Heterogeneity test was performed by the Cochran’s *Q*-statistic [25]. *I* ^*2*^ test was further performed to assess the possibility of heterogeneity among studies [26]. Sensitivity analysis was conducted to assess whether the results were significantly affected via deleting a single study in the current meta-analysis. Publication bias was evaluated by using Deeks funnel plot and publication bias was further confirmed by Egger’s linear regression analysis [26]. All tests in this meta-analysis were two-tailed and *P* < 0.05 was regarded as statistically significant. The meta-analysis was performed using STATA version 12.0 (Stata Corp, College Station, TX, USA) and Meta-Disc version 1.4 (Universidad Complutense, Madrid, Spain).

## Results

### Included studies

**Fig 1:**
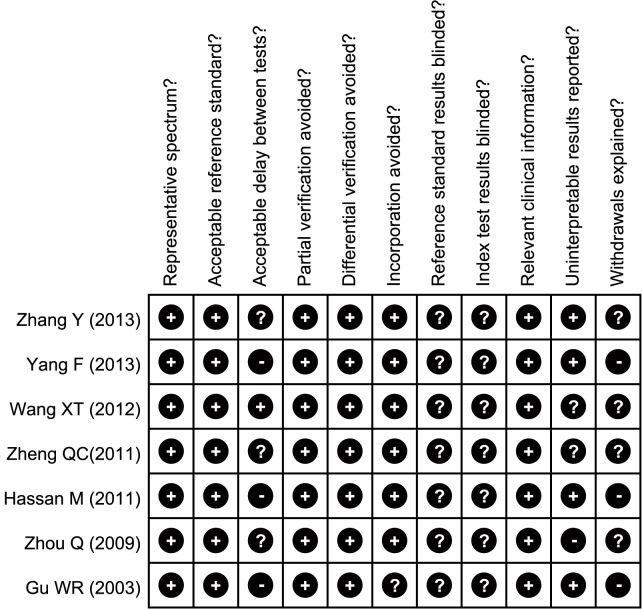
Risk of bias summary: review authors' judgments about each risk of bias item for each included study.

**Fig 2:**
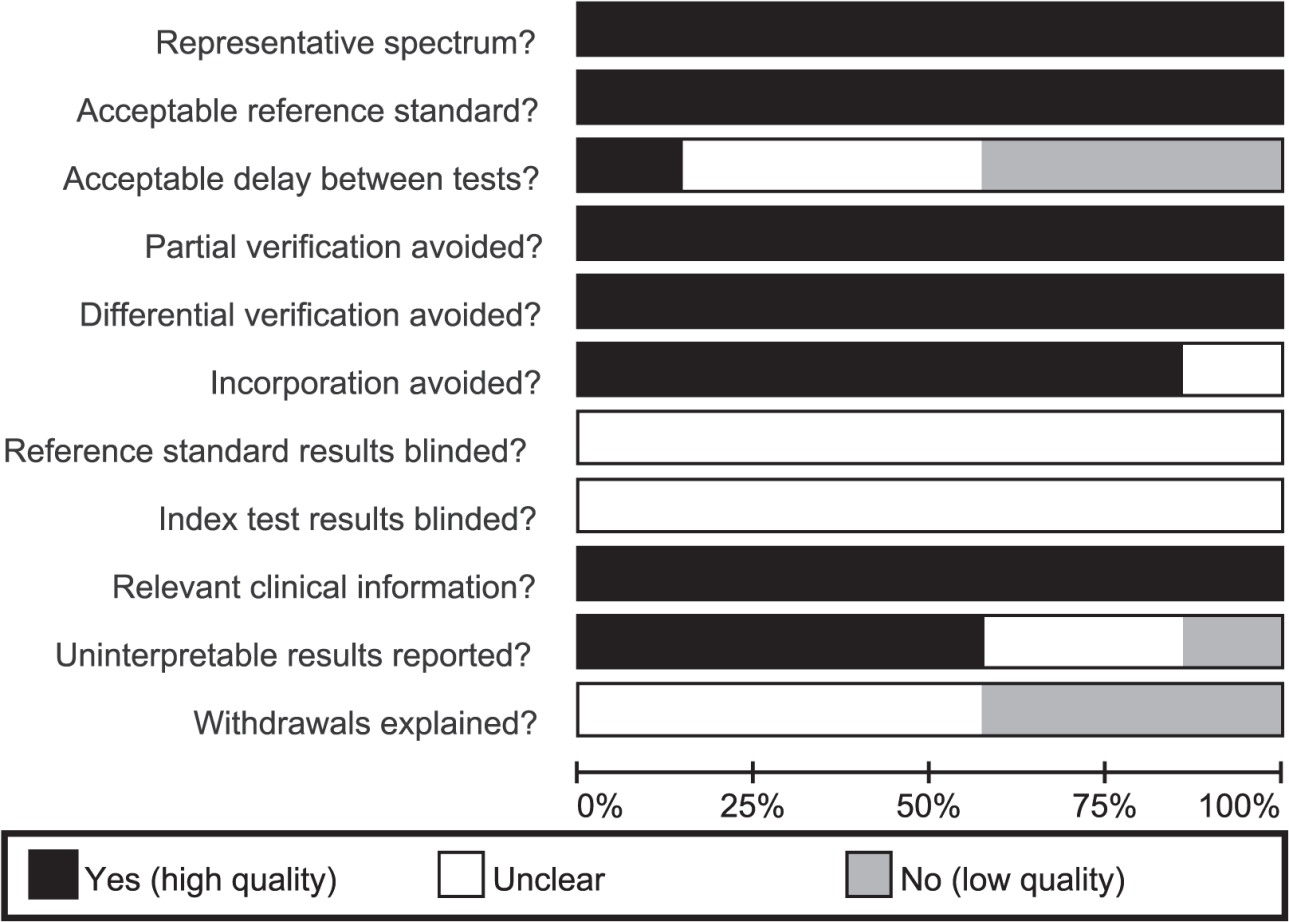
Risk of bias graph: review authors' judgments about each risk of bias item presented as percentages across all included studies.

Initially, 294 studies were retrieved from electronic databases through keyword searches. After excluding duplicates, reviews, letters or meta-analyses, and studies not related to study topics, the remaining studies (n = 141) were reviewed and an additional 134 studies were excluded for being irrelevant to CEUS, or irrelevant to ovarian cancer. A total of 7 studies reported the correlation between CEUS and the diagnosis of ovarian cancer. Finally, these 7 high quality clinical trials, published between 2003 and 2013, were eligible for the present meta-analysis. [19–22,27–29]. A total of 375 ovarian cancer patients were assessed in the seven studies, which included 198 malignant ovarian cancer patients and 177 patients with benign ovarian tumors. The sonographic contrast agent SonoVue was used in six studies, and one study used Levovist. Transabdominal ultrasound was performed in 3 studies and transvaginal ultrasound was done in 4 studies. GE Logiq ultrasound system was used in 4 studies, Philips iU22 ultrasound system in 2 studies and ALT Ultramark ultrasound system was used in the remaining 1 study. Of the included clinical trials, 4 trials used second harmonic imaging (SMI), 3 trials applied pulse inversion harmonic imaging (PIHI). Study subjects, study baseline characteristics and methodological qualities are showed in Table 1 and Fig. 1, 2, respectively.

**Table 1:** Baseline characteristics and methodological quality of all included studies.

First	Year	Sample	size	Age	Tumor size	(cm)	Route	Instrument	Contrast agent	Modality	2×2 table			
author		Malignant	Benign	(years)	Malignant	Benign					TP	FP	FN	TN
Zhang Y [20]	2013	25	23	40.0±11.0	7.3±3.6		Transvaginal	GE Logiq	SonoVue	SMI	24	1	1	22
Yang F [28]	2013	75	31	44.4±12.8	9.1±5.7	7.8±3.3	Transvaginal	Philips iU22	SonoVue	PIHI	70	3	5	28
Wang XT [19]	2012	20	29	40.3±12.2	3.5∼11.2		Transabdominal	Philips iU22	SonoVue	PIHI	19	2	1	27
Zheng QC [29]	2011	14	22	41 (23∼76)	1.2∼6.9	0.8∼9.6	Transabdominal	GE Logiq	SonoVue	SMI	14	2	0	20
Hassan M [27]	2011	22	30	45 (18∼76)	7.5 ± 4.6	7.5±3.9	Transvaginal	GE Logiq	SonoVue	SMI	22	5	0	25
Zhou Q [21]	2009	30	35	42.0±14.0	0.8∼9.6	3.5∼15.2	Transabdominal	GE Logiq	SonoVue	SMI	29	1	1	34
Gu WR [22]	2003	12	7	42.2±10.1	5.8±2.0	5.8±1.5	Transvaginal	ALT Ultramark	Levovist	PIHI	12	2	0	5

Legend: TP—true positive; TN—true negative; FP—false positive; FN—false negative; SMI—second harmonic imaging; PIHI, pulse inversion harmonic imaging.

### Quantitative data synthesis

**Fig 3:**
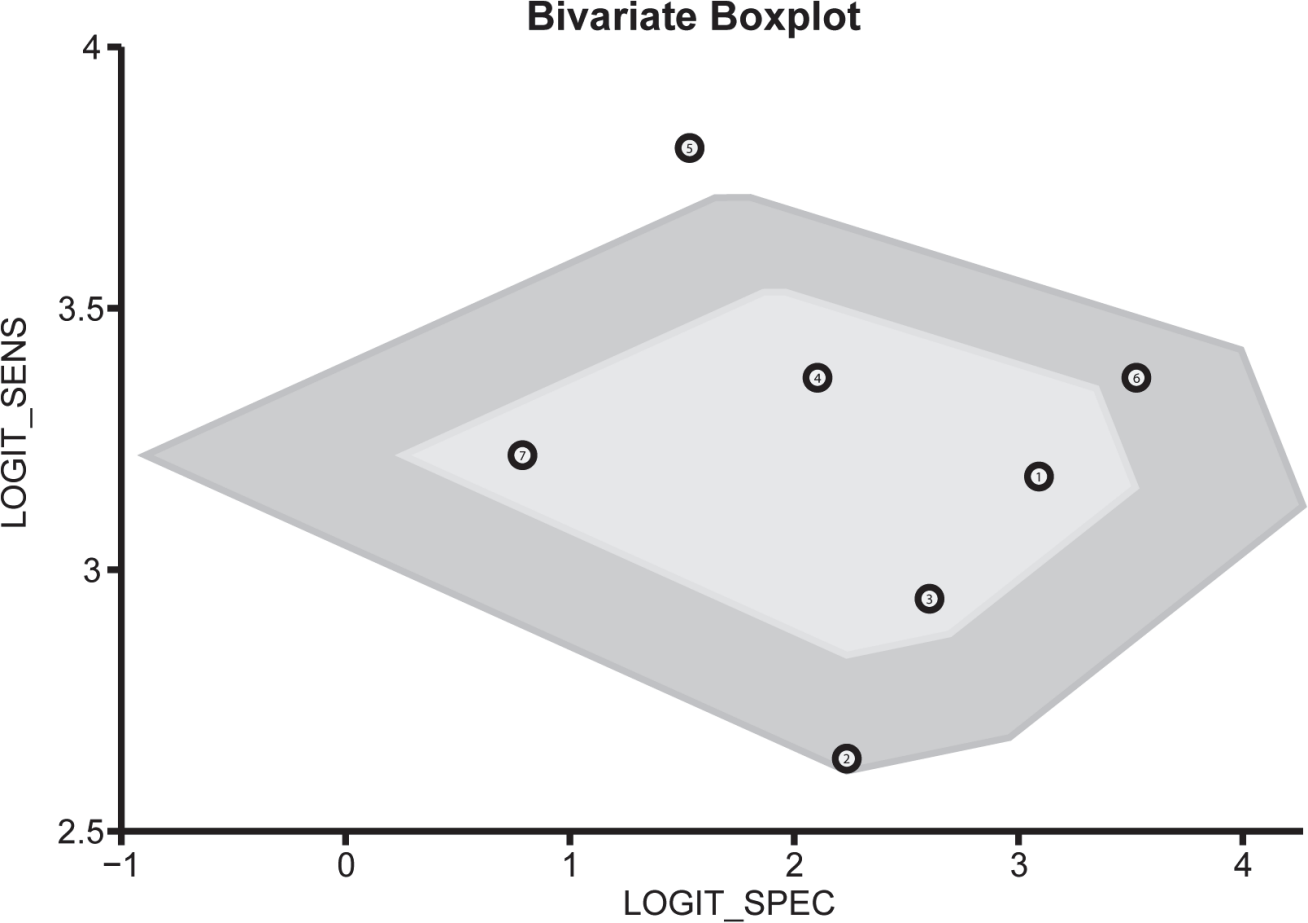
Bivariate Boxplot for contrast-enhanced ultrasonography (CEUS) in differential diagnosis for benign and malignant ovarian tumors.

**Fig 4:**
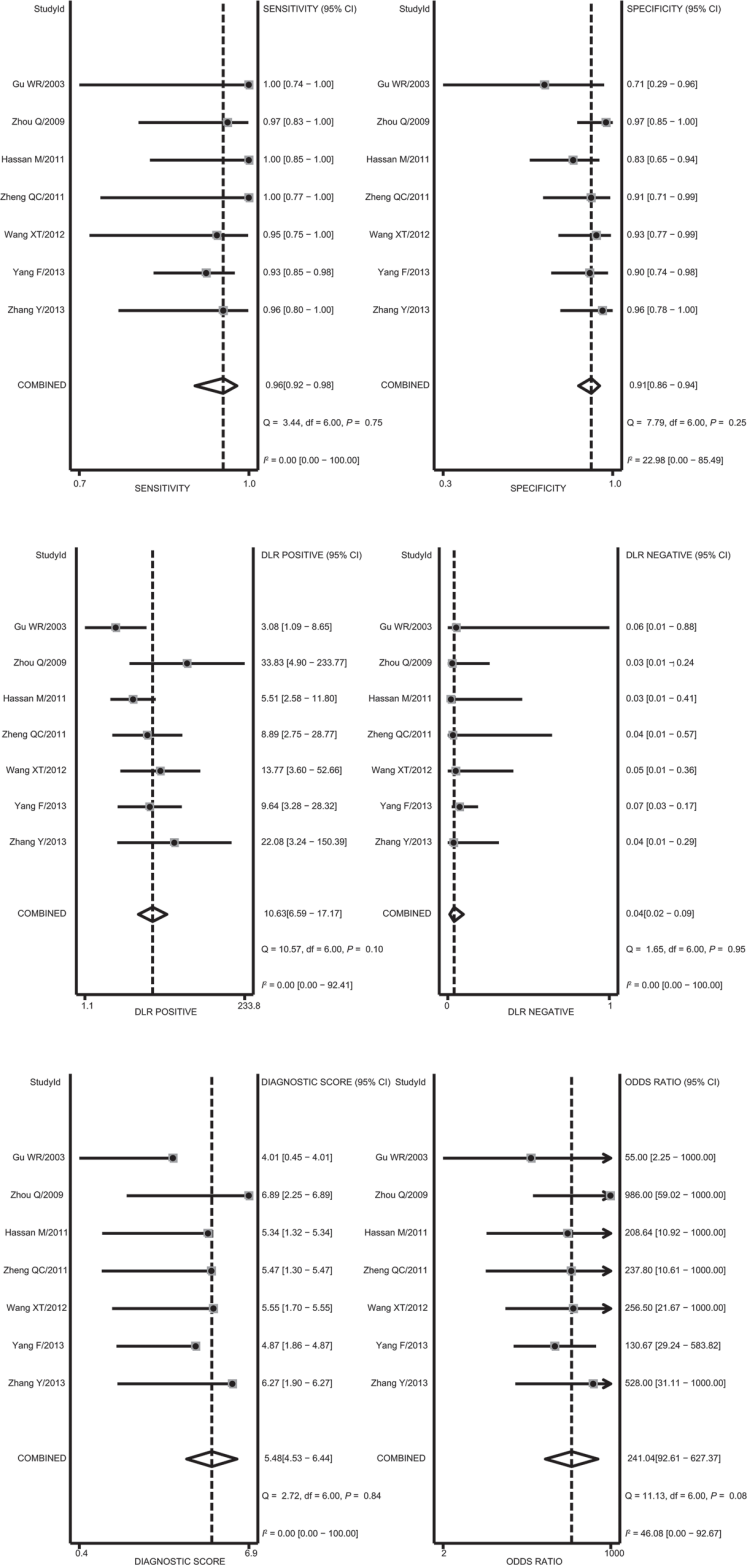
Forest plot of specificity (Spe), sensitivity (Sen), positive and negative likelihood ratios (LR^+^/LR^−^), and diagnostic odds ratio (DOR) with their 95%Cis for contrast-enhanced ultrasonography (CEUS) in differential diagnosis for benign and malignant ovarian tumors.

**Fig 5:**
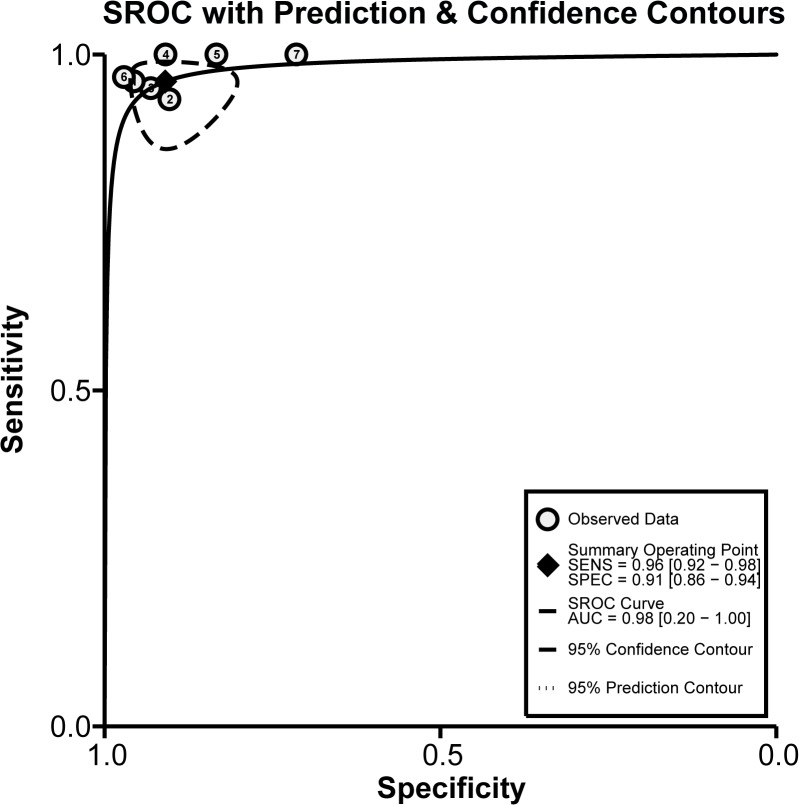
The summary receiver operating characteristic (SROC) curve for contrast-enhanced ultrasonography (CEUS) in differential diagnosis for benign and malignant ovarian tumors.

**Fig 6:**
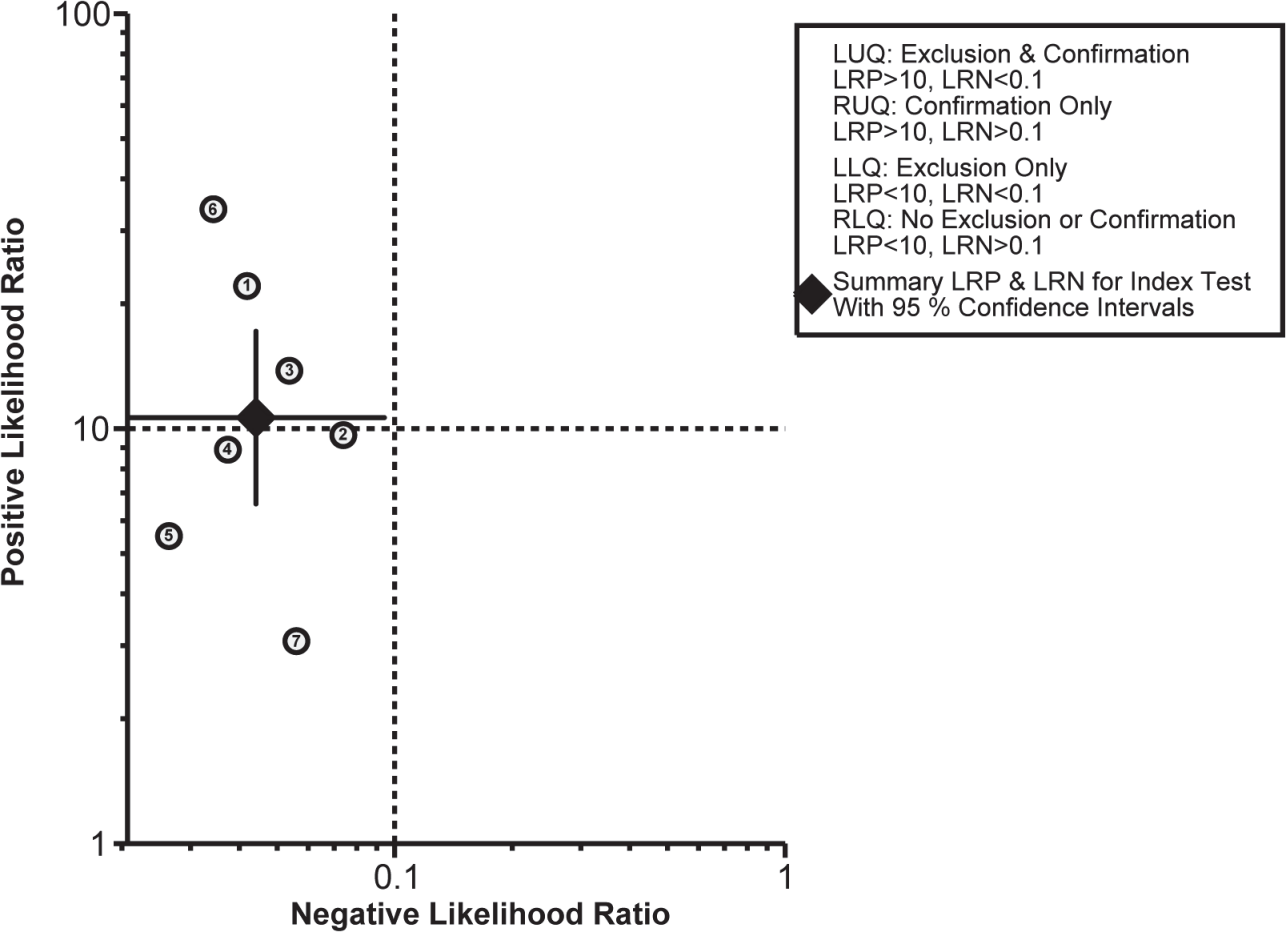
Likelihood ratios plot for contrast-enhanced ultrasonography (CEUS) in differential diagnosis for benign and malignant ovarian tumors.

**Fig 7:**
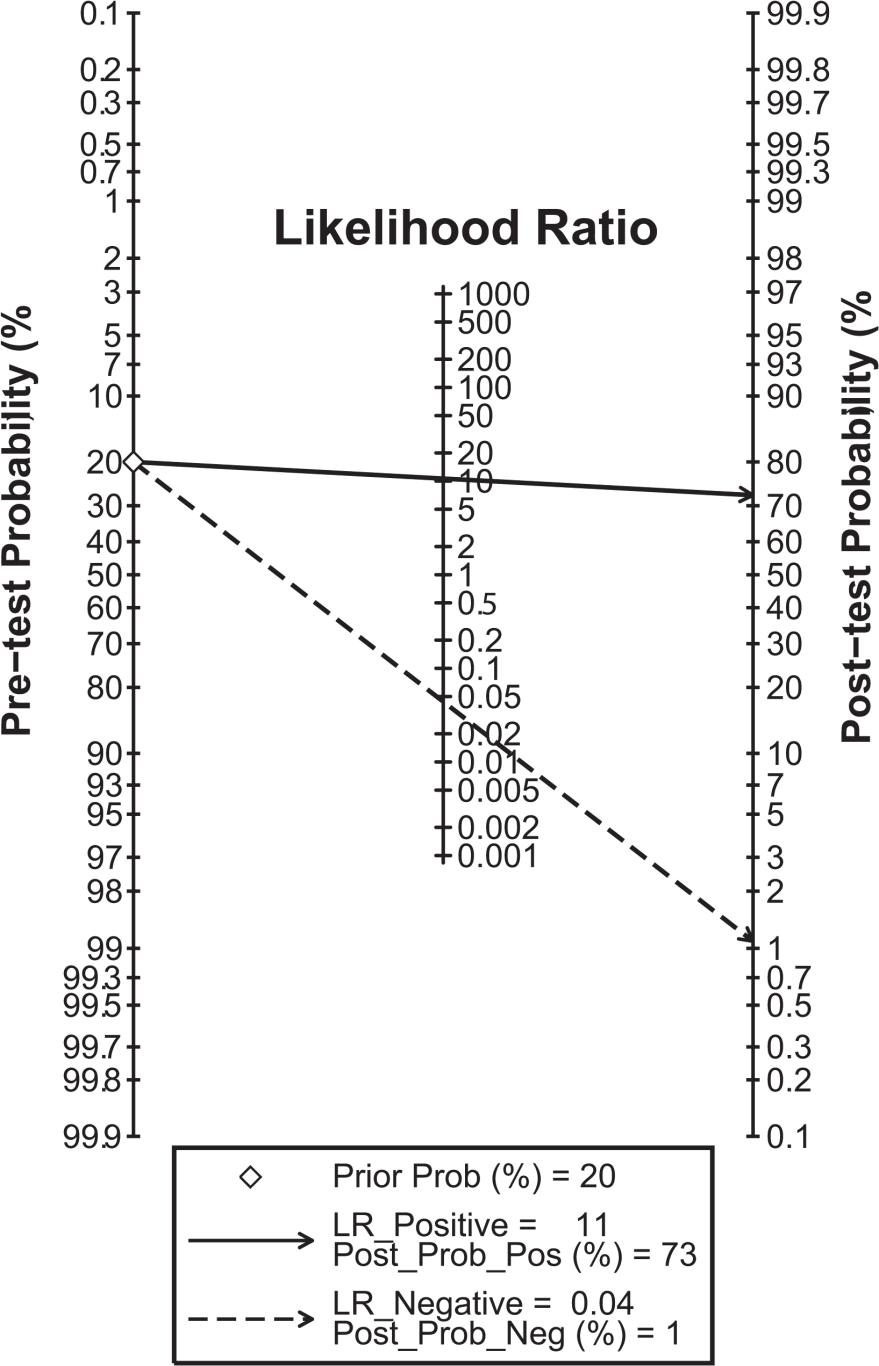
Fagan’s Nomogram plot for contrast-enhanced ultrasonography (CEUS) in differential diagnosis for benign and malignant ovarian tumors.

**Fig 8:**
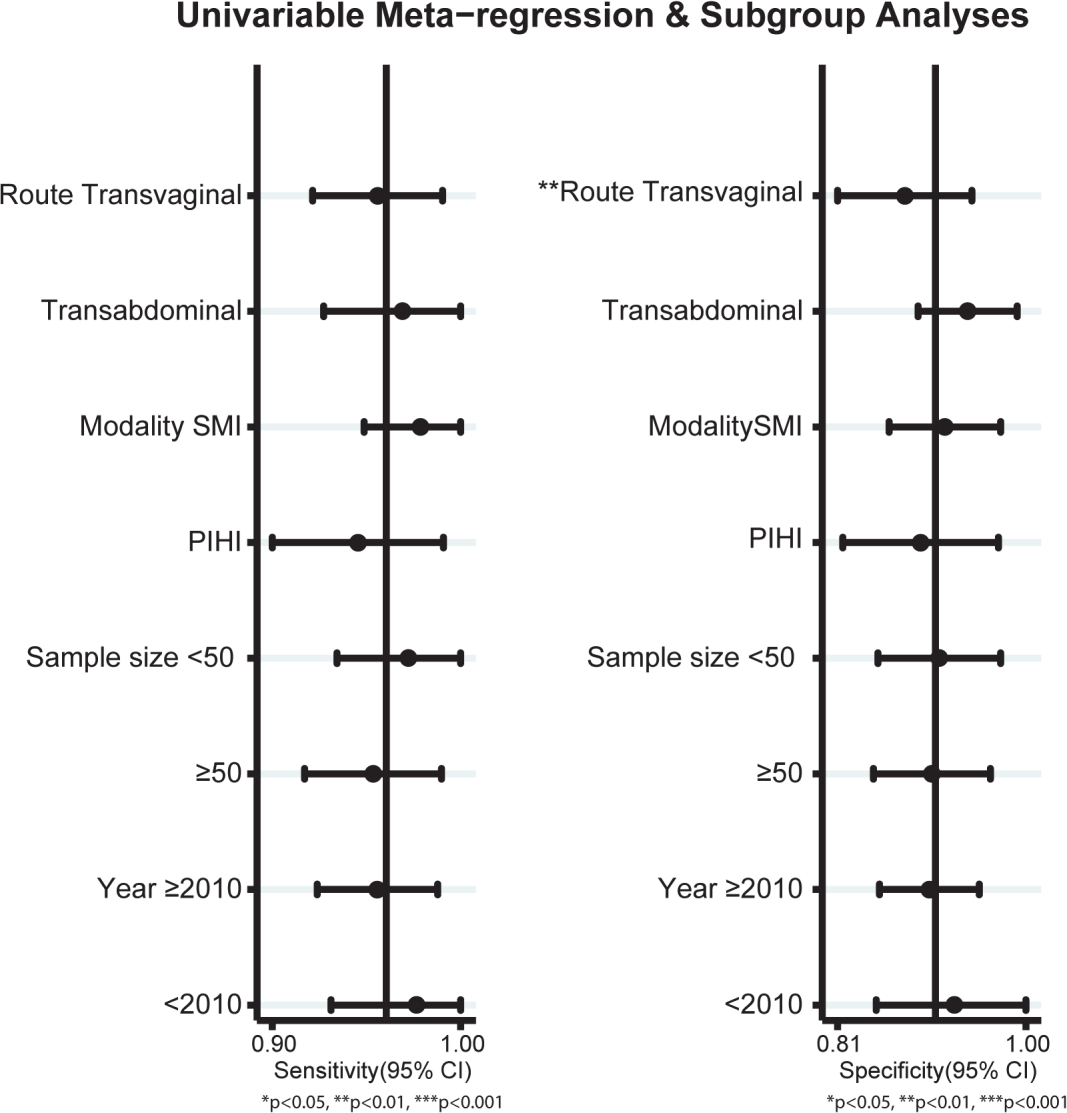
Meta-regression plot for contrast-enhanced ultrasonography (CEUS) in differential diagnosis for benign and malignant ovarian tumors.

The diagnostic accuracy of CEUS in discriminating between malignant ovarian cancer and benign ovarian tumor was measured as pooled Sen, Spe, LR^+^, LR^−^ and DOR (Fig. 3). Random effects model was used due to the existence of significant heterogeneity among studies(Fig. 4). Our meta-analysis revealed that CEUS enhancement pattern of malignant ovarian cancer was centrifugal stability enhancement, and benign ovarian tumors showed an annular enhancement, and the pooled Sens was 0.96 (95%CI = 0.92∼0.98) and the pooled Spes was 0.91 (95%CI = 0.86∼0.94) in malignant ovarian cancers. There was no significant association (*r* = −0.505, *P* = 0.414) between sensitivity and specificity, which indicated the absence of threshold effect. In addition, we observed that the pooled LR^+^ and LR^−^ were 10.63 (95%CI = 6.59∼17.17) and 0.04 (95%CI = 0.02∼0.09), respectively. The pooled DORs of CEUS in qualitative diagnosis of ovarian cancer was 241.04 (95%CI = 92.61∼627.37). The results were plotted as a symmetrical SROC curve and the corresponding AUC was 0.98 (95%CI = 0.20∼1.00), suggesting a moderate diagnostic value (Fig. 5). Pooled likelihood ratio analysis indicated the limited diagnosis value of CEUS in differential diagnosis of benign and malignant ovarian tumors (Fig. 6). The results of Fagan’s Nomogram indicated that pretest probability ratio is 20%, the post-test probability for LR+ is 73%, for LR− is 1% (Fig. 7). Meta-regression analysis showed that, route, modality, sample size and publication year were not the main factors influencing the heterogeneity for Sen; while for Spe, route may be the main source of heterogeneity (Table 2 and Fig. 8).

**Table 2:** Meta-regression analyses of potential source of heterogeneity.

Parameter	category	Number of studies	Sensitivity	p1	Specificity	p2
Route	Transvaginal	4	0.96(0.92–0.99)	0.28	0.88(0.81–0.95)	0
	Transabdominal	3	0.97(0.93–1.00)		0.94(0.89–0.99)	
Modality	SMI	4	0.98(0.95–1.00)	0.63	0.92(0.86–0.97)	0.18
	PIHI	3	0.94(0.90–0.99)		0.89(0.82–0.97)	
Sample size	< 50	4	0.97(0.93–1.00)	0.94	0.91(0.85–0.97)	0.05
	≥ 50	3	0.95(0.92–0.99)		0.91(0.85–0.96)	
Year	≥ 2010	5	0.96(0.92–0.99)	0.71	0.90(0.85–0.95)	0.11
	< 2010	2	0.98(0.93–1.00)		0.93(0.85–1.00)	

SMI—second harmonic imaging; PIHI, pulse inversion harmonic imaging.

### Sensitivity Analysis and Publication Bias

**Fig 9:**
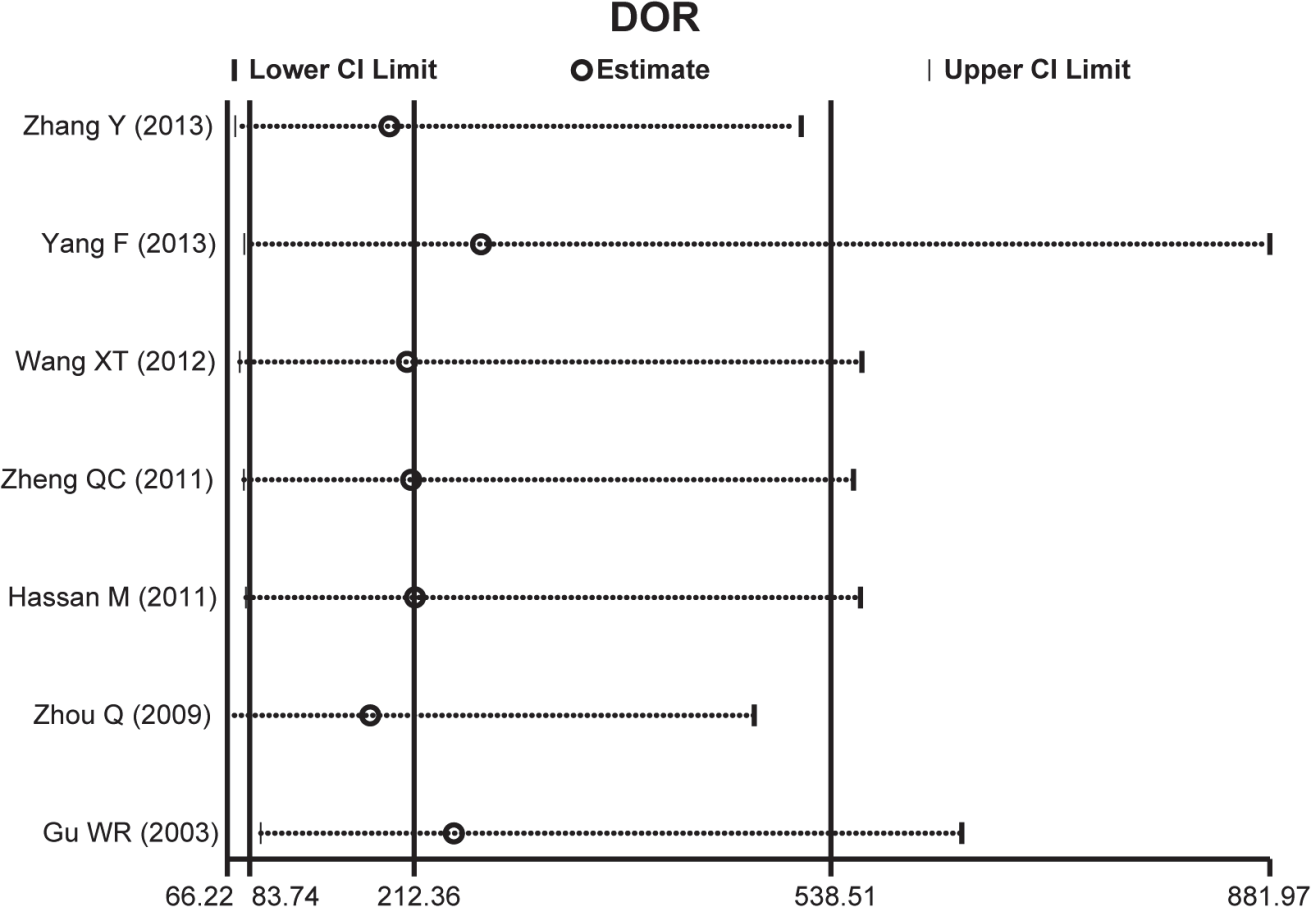
Sensitivity analysis for contrast-enhanced ultrasonography (CEUS) in differential diagnosis for benign and malignant ovarian tumors.

**Fig 10:**
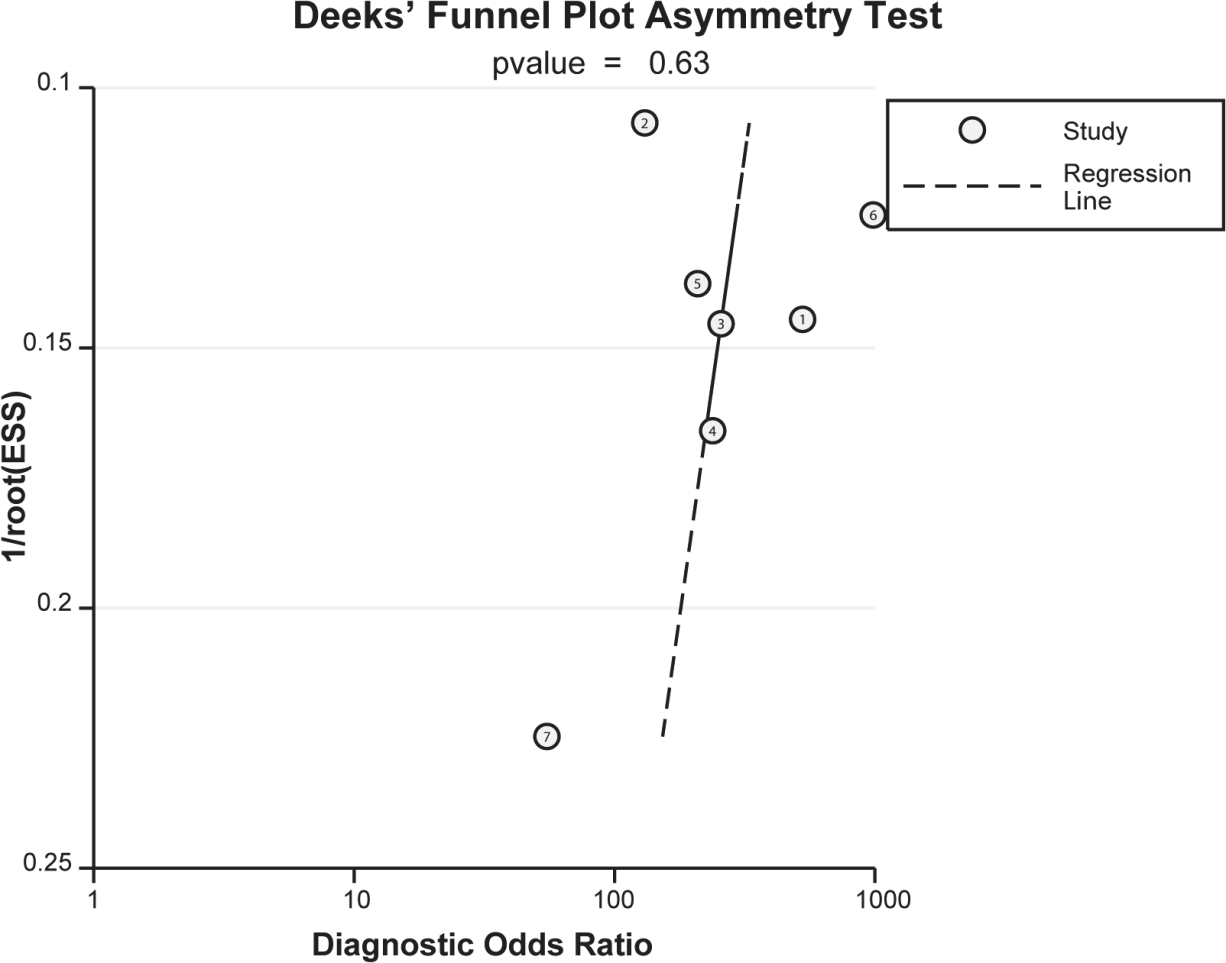
Deeks funnel plot for contrast-enhanced ultrasonography (CEUS) in differential diagnosis for benign and malignant ovarian tumors.

Sensitivity analysis demonstrated that no single study had marked effect on the overall effect sizes (Fig. 9). The included angle between regression line and DOR axis was close to 90° in deeks funnel plot, and illustrated minimal publication bias in current meta-analysis (Fig. 10). Egger’s linear regression analysis further confirmed that no publication bias existed among the included studies (t = −0.52, P = 0.626).

## Discussion

CEUS requires the administration of ultrasound contrast agents, which generally containsgas-filled micro-bubbles with a stabilized shell, small enough to pass through the blood vessels when administered intravenously [30]. Technological advances have allowed rapid expansion in the clinical application of CEUS [31], and recently, CEUS was used to differentiate benign ovarian tumors from malignant ovarian cancers [32]. CEUS has the capability to clearly display microvascular blood flow in tumors, and can accurately evaluate the sequence and intensity of tumor perfusion and vascularity. Not surprisingly, CEUS is already regarded as a superior technique for diagnosis of malignant ovarian cancers at early stages [33]. In addition, CEUS can detect the differences in microcirculations between benign and malignant tumors, and is able to qualitatively diagnose different types of lesions. Therefore, CEUS could become a valuable diagnostic tool for screening high-risk populations for ovarian cancers [21].

The technical performance and detection accuracy of CEUS in differential diagnosis of benign and malignant ovarian tumors was carefully evaluated in this meta-analysis. We extracted data from seven independent high quality studies containing a total of 198 malignant ovarian cancer patients and 177 benign ovarian tumor patients. Our results showed that the pooled Sen, Spe and DOR of CEUS in diagnosing ovarian cancer were 96%, 91% and 212.18, respectively. These results demonstrated the high diagnostic accuracy and detection efficiency of CEUS for ovarian cancer. One possible explanation for the high specificity could be that the CEUS contrast agent inherently prevents false sonographic images and the micro-bubbles offers superior technical capabilities in differentiating ovarian cancers even from small blood vessels, slow blood flows and similar acoustic properties, thereby resulting in improved diagnostic accuracy [13,34]. Our findings showed no significant relationship between Sen and Spe, revealing no evidence of threshold effect within the selected studies, which refers to sudden and radical change after a quantitative limit is exceeded. Importantly, the existence of a threshold divides a group into two sub-groups, one above the threshold and one below the threshold [35]. Meta-regression analyses showed the differences in sample size, route and instrument type did not influence the CEUS diagnostic accuracy, indicating that CEUS exhibited a high diagnostic performance in different subgroups. Furthermore, our results found no direct evidence of publication bias. Our results are partly consistent with previous studies, and strongly support the high accuracy of CEUS in differential diagnosis of benign and malignant ovarian tumors.

Our present meta-analysis is the first to focus on the accuracy of CEUS in distinguishing malignant ovarian tumors from benign lesions. However, our study has limitations. First, due to the relatively small sample size (n = 7, with a total sample size of 375), insufficient statistical power may restrict our ability to evaluate the accuracy of CEUS. In addition, this meta-analysis was retrospective, which may strongly influence the results due to selection bias. Third, important data from the present publications and original authors was unavailable, therefore, data extraction was difficult, which may have adversely effected the clinical assessment of CEUS for its value indiagnosis of ovarian cancer. Fourth, we did not compare the diagnostic performance of CEUS with other imaging tools used for distinguishing between benign and malignant ovarian tumors.

In conclusion, our results strongly support the high diagnostic value of CEUS in differential diagnosis of benign and malignant tumors in ovarian cancer patients. CEUS may be an effective tool for detection of early stage ovarian cancers. However, further studies are required in a larger sample size and in diverse ethnic populations to fully confirm our findings.

## Supporting Information

S1 PRISMA Checklist (DOC)

## References

[1] Fan T, Zhao Q, Chen JJ, Chen WT, Pearl ML. Clinical significance of circulating tumor cells detected by an invasion assay in peripheral blood of patients with ovarian cancer. Gynecol Oncol, 2009; 112: 185–191. 10.1016/j.ygyno.2008.09.021 18954898 PMC2606929

[2] Banys M, Solomayer EF, Becker S, Krawczyk N, Gardanis K, Staebler A, et al Disseminated tumor cells in bone marrow may affect prognosis of patients with gynecologic malignancies. Int J Gynecol Cancer, 2009; 19: 948–952. 10.1111/IGC.0b013e3181a23c4c 19574790

[3] Drescher CW, Hawley S, Thorpe JD, Marticke S, McIntosh M, Gambhir SS, et al Impact of screening test performance and cost on mortality reduction and cost-effectiveness of multimodal ovarian cancer screening. Cancer Prev Res (Phila), 2012; 5: 1015–1024. 10.1158/1940-6207.CAPR-11-0468 22750949 PMC3729263

[4] Menon U, Gentry-Maharaj A, Hallett R, Ryan A, Burnell M, Sharma A, et al Sensitivity and specificity of multimodal and ultrasound screening for ovarian cancer, and stage distribution of detected cancers: results of the prevalence screen of the UK Collaborative Trial of Ovarian Cancer Screening (UKCTOCS). Lancet Oncol, 2009; 10: 327–340. 10.1016/S1470-2045(09)70026-9 19282241

[5] Tsubamoto H, Sonoda T, Yamasaki M, Inoue K. Impact of combination chemotherapy with itraconazole on survival of patients with refractory ovarian cancer. Anticancer Res, 2014; 34:2481–2487. 24778064

[6] Milne RL, Gaudet MM, Spurdle AB, Fasching PA, Couch FJ, Benítez J, et al Assessing interactions between the associations of common genetic susceptibility variants, reproductive history and body mass index with breast cancer risk in the breast cancer association consortium: a combined case-control study. Breast Cancer Res, 2010; 12: R110 10.1186/bcr2797 21194473 PMC3046455

[7] Tang HS, Feng YJ, Yao LQ. Angiogenesis, vasculogenesis, and vasculogenic mimicry in ovarian cancer. Int J Gynecol Cancer, 2009; 19: 605–610. 10.1111/IGC.0b013e3181a389e6 19509557

[8] Pickles MD, Manton DJ, Lowry M, Turnbull LW. Prognostic value of pre-treatment DCE-MRI parameters in predicting disease free and overall survival for breast cancer patients undergoing neoadjuvant chemotherapy. Eur J Radiol, 2009; 71: 498–505. 10.1016/j.ejrad.2008.05.007 18572340

[9] Quaia E. Assessment of tissue perfusion by contrast-enhanced ultrasound. Eur Radiol, 2011; 21: 604–615. 10.1007/s00330-010-1965-6 20927527

[10] Wilson SR, Greenbaum LD, Goldberg BB. Contrast-enhanced ultrasound: what is the evidence and what are the obstacles? AJR Am J Roentgenol, 2009; 193: 55–60. 10.2214/AJR.09.2553 19542395

[11] Rafaelsen SR, Jakobsen A. Contrast-enhanced ultrasound vs multidetector-computed tomography for detecting liver metastases in colorectal cancer: a prospective, blinded, patient-by-patient analysis. Colorectal Dis, 2011; 13: 420–425. 10.1111/j.1463-1318.2010.02288.x 20412096

[12] Bolondi L, Correas JM, Lencioni R, Weskott HP, Piscaglia F. New perspectives for the use of contrast-enhanced liver ultrasound in clinical practice. Dig Liver Dis, 2007; 39(2): 187–195. 17208526 10.1016/j.dld.2006.08.008

[13] Fleischer AC, Lyshchik A, Jones HW Jr, Crispens M, Loveless M, Andreotti RF, et al Contrast-enhanced transvaginal sonography of benign versus malignant ovarian masses: preliminary findings. J Ultrasound Med, 2008; 27: 1011–1018; quiz 1019–1021. 18577664 10.7863/jum.2008.27.7.1011

[14] Mironov S, Akin O, Pandit-Taskar N, Hann LE. Ovarian cancer. Radiol Clin North Am, 2007; 45: 149–166. 17157627 10.1016/j.rcl.2006.10.012

[15] Rubatt JM, Darcy KM, Hutson A, Bean SM, Havrilesky LJ, Grace LA, et al Independent prognostic relevance of microvessel density in advanced epithelial ovarian cancer and associations between CD31, CD105, p53 status, and angiogenic marker expression: A Gynecologic Oncology Group study. Gynecol Oncol, 2009; 112: 469–474. 10.1016/j.ygyno.2008.11.030 19135712

[16] Gay F, Pierucci F, Zimmerman V, Lecocq-Teixeira S, Teixeira P, Baumann C, et al Contrast-enhanced ultrasonography of peripheral soft-tissue tumors: Feasibility study and preliminary results. Diagn Interv Imaging, 2012; 93: 37–46. 10.1016/j.diii.2011.11.007 22277709

[17] Sorelli PG, Cosgrove DO, Svensson WE, Zaman N, Satchithananda K, Barrett NK, et al Can contrast-enhanced sonography distinguish benign from malignant breast masses? J Clin Ultrasound, 2010; 38: 177–181. 10.1002/jcu.20671 20146214

[18] Hastings JM, Morris KD, Allan D, Wilson H, Millar RP, Fraser HM, et al Contrast imaging ultrasound detects abnormalities in the marmoset ovary. Am J Primatol, 2012; 74: 1088–1096. 10.1002/ajp.22063 22890799

[19] Wang XT, Wang R, Cui JH, Zhu QY. Applications of contrast-enhanced ultrasonography in diagnosis and differential of ovarian masses. Jiangsu Med J, 2012; 38: 88–90.

[20] Zhang Y, Zhou J, Li MX, Luo ZJ. Clinical value of contrast-enhanced ultrasonography in the qualitative diagnosis of ovarian cancer. J Clin Ultrasound in Med, 2013; 15: 403–405.

[21] Zhou Q, Liu BL, Jiang J, Lei XY. Value of color Doppler ultrasonography, contrast-enhanced ultrasound and serum CA-125 detection in differential diagnosis of ovarian masses. J Southern Med Univ, 2009; 29: 2007–2009. 19861251

[22] Gu WR, Feng YJ, Zhang JH. Efficacy of Levovist in color Doppler ultrasonography of benign and malignant ovarian tumors. Chin J Ultrasonography, 2003; 12: 21–24.

[23] Whiting PF, Weswood ME, Rutjes AW, Reitsma JB, Bossuyt PN, Kleijnen J. Evaluation of QUADAS, a tool for the quality assessment of diagnostic accuracy studies. BMC Med Res Methodol, 2006; 6: 9 16519814 10.1186/1471-2288-6-9 PMC1421422

[24] Hamza TH, Arends LR, van Houwelingen HC, Stijnen T. Multivariate random effects meta-analysis of diagnostic tests with multiple thresholds. BMC Med Res Methodol, 2009; 9: 73 10.1186/1471-2288-9-73 19903336 PMC2787531

[25] Zintzaras E, Ioannidis JP. HEGESMA: genome search meta-analysis and heterogeneity testing. Bioinformatics, 2005; 21: 3672–3673. 15955784 10.1093/bioinformatics/bti536

[26] Peters JL, Sutton AJ, Jones DR, Abrams KR, Rushton L. Comparison of two methods to detect publication bias in meta-analysis. JAMA, 2006; 295: 676–680. 16467236 10.1001/jama.295.6.676

[27] Hassan M, Teh R. Applications of contrast-enhanced ultrasonography in diagnosis and differential of ovarian cancer. Medical J Chinese People's Health. 2011; 23: 2089–2040.

[28] Yang F, Yang TZ, Luo H, Song B. Diagnostic value of contrast-enhanced ultrasonography on ovarian tumors. J Sichuan Univer (Med Sci Edition), 2013; 44: 424–428. 23898527

[29] Zheng QC, Li P, Wang YH, Liang NN, Cai HB. Significance of contrast-enhanced ultrasonography in differential diagnosis of benign and malignant ovarian tumors. J Clin Ultrasound in Med, 2011; 13: 301–303.

[30] Dindyal S, Kyriakides C. Ultrasound microbubble contrast and current clinical applications. Recent Pat Cardiovasc Drug Discov, 2011; 6: 27–41. 21222650 10.2174/157489011794578446

[31] Balleyguier C, Opolon P, Mathieu MC, Athanasiou A, Garbay JR, Delaloge S, et al New potential and applications of contrast-enhanced ultrasound of the breast: Own investigations and review of the literature. Eur J Radiol, 2009; 69: 14–23. 10.1016/j.ejrad.2008.07.037 18977102

[32] Xing W, Zhigang W, Bing H, Haitao R, Pan L, Chuanshan X, et al Targeting an ultrasound contrast agent to folate receptors on ovarian cancer cells: feasibility research for ultrasonic molecular imaging of tumor cells. J Ultrasound Med, 2010; 29: 609–614. 20375379 10.7863/jum.2010.29.4.609

[33] Fleischer AC, Lyshchik A, Jones HW 3rd, Crispens MA, Andreotti RF, Williams PK, et al Diagnostic parameters to differentiate benign from malignant ovarian masses with contrast-enhanced transvaginal sonography. J Ultrasound Med, 2009; 28: 1273–1280. 19778872 10.7863/jum.2009.28.10.1273

[34] Smeenge M, Mischi M, Laguna Pes MP, de la Rosette JJ, Wijkstra H. Novel contrast-enhanced ultrasound imaging in prostate cancer. World J Urol, 2011; 29: 581–587. 10.1007/s00345-011-0747-3 21847656 PMC3189413

[35] Lohmueller KE, Pearce CL, Pike M, Lander ES, Hirschhorn JN. Meta-analysis of genetic association studies supports a contribution of common variants to susceptibility to common disease. Nat Genet, 2003; 33: 177–182. 12524541 10.1038/ng1071

